# Demystifying the effect of social media eWOM on revisit intention post-COVID-19: an extension of theory of planned behavior

**DOI:** 10.1186/s43093-022-00161-5

**Published:** 2022-10-08

**Authors:** Mohd Azhar, Ruksar Ali, Sheeba Hamid, Mohd Junaid Akhtar, Mohd Nayyer Rahman

**Affiliations:** Department of Commerce, Aligarh Muslim University, Aligarh, 202002 India

**Keywords:** Theory of planned behavior, Social media, eWOM, Revisit intention, Destination image, COVID-19

## Abstract

The present study intends to unwrap the influence of social media electronic word of mouth (eWOM) on revisit intention post-COVID-19 applying the theory of planned behavior (TPB). Two additional constructs, viz., eWOM and destination image, have been undertaken in the present study to enhance the robustness of the TPB model. An online questionnaire was employed to collect data, and the research relied upon 301 correct and useable responses. The survey's population includes potential tourists who intend to revisit India post-COVID-19. SPSS 20 and AMOS 22.0 were used to analyze the data. The posited model was validated using confirmatory factor analysis (CFA) and structural equation modeling (SEM). The findings indicate that all of the constructs under study, namely "electronic word of mouth (eWOM), destination image (DI), attitude (ATT), subjective norm (SN), and perceived behavioral control (PBC)," significantly and positively influence "tourists' revisit intention (RI)" post-COVID-19. These constructs explained approximately 71% (*R*^2^ = 0.709) of the variance in the revisit intention post-COVID-19. A number of theoretical and practical implications can be delineated to make recommendations to the ministry of tourism, tour and travel agencies, central and state government-owned tourism departments, marketers and promoters of travel destinations. The distinctiveness of the present study lies in the fact that it measures the influence of eWOM on revisit intention post-COVID-19 in the Indian context by incorporating destination image with the TPB model.

## Introduction

The tourism industry is widely recognized as a substantial driver of economic growth as well as a primary source of wealth creation, livelihoods and income [99, 112]. It has grown and diversified tremendously over the last several years and has become one of the fastest-growing economic sectors in the world [8, 124]. Tourism in the twenty-first century is intrinsically linked to the development, expansion, growth and sustenance of tourists' destinations [1]. These nuances have facilitated the emergence of the tourism industry as a vital catalyst for socio-economic development [23]. But the abruption of the COVID-19 pandemic severely affected the world economy [160] and the global environment [159]. This resultantly hindered the growth of travel and tourism-related activities and led it to a halting stage [13, 69]. The travel and tourism industry was one of the most severely impacted industries throughout the world [90], and India was not an exception in this regard [69]. COVID-19 restrictions seriously impacted global economic growth [161]. According to a report by the World Bank, nearly 47.7 million tourism-related jobs in South Asia were impacted by the COVID-19 pandemic. This report further states that the Indian economy witnessed a potential US $ 43.4 billion loss in GDP [164]. A report of the United Nations Conference on Trade and Development (UNCTAD) states that international tourists' arrival in developing countries dropped down to 80–90% in 2020 compared to the previous years [150]. It is projected that it would take two years to go back to the levels that were before the outbreak [140]. Numerous scholars have taken an interest in developing and carrying out plans and actions that will bring the travel and tourism sector back to its normal (pre-pandemic) state or to a state that is in some way superior to that normal one [142]. In this regard, the present study discusses various antecedents and their consequences on revisit intention post-COVID-19.

Undoubtedly, innumerable studies, particularly in the last decade, have discussed revisit intention and its antecedents [14, 104, 135]. Business expansion and survival in the tourism industry often depend on tourists' revisit intention [115]. For companies to flourish and succeed, revisit intention is highly valued [1]. The key point in understanding tourist revisit intention lies in the fact that predicting travel intention cuts down unnecessary marketing and promotional expenses that boost the tourism industry's profitability [69, 104]. Successful destination marketing may depend on the ability to predict revisit intention, which may provide a cost advantage over competitors [23]. In addition, it helps the tourism industry grow and thrive [12, 141] and is often considered the most important factor in effective destination marketing [23, 104]. Given that the tourism industry is service-oriented, it is responsive to tourists' experiences and overall satisfaction with the products and services they receive [55]. Therefore, it has become quite indispensable to study revisit intention post-COVID-19.

The world faced a generationally exceptional economic and health crisis in the form of the recent COVID-19 pandemic. But the loosening of restrictions post-COVID-19 has accelerated global economic activities [158]. And the travel is slowly gaining its pace again. But making travel-related decisions is highly complicated and is influenced by a number of factors [56], viz. "attitudes, subjective norm and perceived behavioral control" [28]. According to tourism literature, the destination image is the most important factor that plays a key role in determining to revisit intention [128, 173] and has a considerable influence on tourists' behavior [11, 162]. Choosing a tourist destination is undeniably a complex, uncertain and muddled procedure [136]. Researchers have also stated that the theory of planned behavior (TPB) has become a vital aspect, especially in planning tourism-related destinations be it domestic or international [42, 81]. Many tourism studies have embraced the TPB model as one of the most potent means for testing tourists' overall behavioral intention post-COVID [69, 103, 117]. Therefore, in the present study, TPB has been used to predict revisit intention comprising key components, attitude, subjective norm and perceived behavioral control along with additional constructs, viz. destination image and eWOM. Moreover, the influence of eWOM on revisit intention has also been studied.

Social media has emerged as the most prominent medium for exchanging information related to travel, tourism and hospitality in the recent past [39]. The fast expansion and broad acceptance of social media have piqued the attention of tourist and leisure marketers [100]. Nowadays, social media sees a resurgence, with hundreds of individuals posting their thoughts on their travel experiences on the platform [30, 37, 59]. Facebook, Twitter, Instagram, YouTube, etc., have become important add-ons to share travel-related views and experiences [59, 132]. Personal blogs have been a significant source of information about travel [101]. Social media has proved a very strong medium of information during the recent pandemic, COVID-19 [92, 169], be it related to routine discussions, crisis-related communication or purchase and travel decisions. Because of wide interactivity, ubiquity and mobility, social media has emerged as the most popular means of eWOM communication [30]. eWOM plays a crucial role in building a positive or negative image of any destination and helps consumers in decision-making [30].

Despite the increasing use of social media by prospective decision-makers, it is still required to understand the phenomena of eWOM in tourism, specifically after COVID-19. Therefore, the present study reinvestigates the influence of social media eWOM on revisit intention post-COVID-19. Moreover, it measures various factors influencing tourist intention to revisit by implementing the theory of planned behavior. Several studies have been performed on eWOM and social media in the tourism context. Still, fewer studies have tested the influence of eWOM on revisit intention and that too during any disaster, natural calamity, health crisis or the recent COVID-19 pandemic. The newness of this study lies in the fact that it has incorporated two additional constructs, viz. eWOM and destination image, into the TPB model to assess the robustness of the model and re-examine the impact of eWOM on revisit intention post-COVID-19. These additional constructs are very relevant while assessing revisit intention post-COVID-19.

## Theoretical background

It is no more an enigma that social media has become one of the finest avenues for exchanging eWOM among travelers [30]. It is important to recognize that social media encompasses an extensive array of specific media kinds, such as "blog posts, message boards, review sites, social networking sites, messaging applications, video sharing sites, etc." [59]. These platforms provide huge content on tourism, such as destination images, video content and personal reviews on various tourism destinations [30, 59, 84]. Travel consumer research shows that the role of social media is the highest in the pre-trip phase [39]. Consumers use reviews and other content to gain insights and analyze alternatives to reduce their choices [60]. Previous research has shown that travel plans are susceptible to social media content. It is an essential part of their travel searches, even if they are not intentionally looking for social media sources [166].

The worldwide travel sector has nearly ground to a standstill due to the COVID-19 pandemic. Consequently, destinations throughout the world have employed well-established crisis responses in an effort to repair their reputations, regain visitors' confidence and lure back international visitors [165]. Marketers in the tourism industry, like their counterparts in other sectors, must use a variety of media to reassure consumers that their locations are safe and appealing during times of crisis [24, 125, 165]. During COVID-19, the influence of social media was more intense on tourists' behavior, perception and attitude because of extensive COVID-19-related information [27]. Various factors influence individual choices of destination and affect how one perceives eWOM and social media-generated content regarding any destination. Predicting behavioral intention due to eWOM using TPB is well researched by previous studies [30, 96, 113]. Personal opinions and reviews about a destination (WOM) significantly influence one's behavioral beliefs (attitude), normative beliefs (subjective norm) and control beliefs (perceived behavioral control) [84]. This forms a basis for connecting eWOM and TPB to analyze revisit intention. Thus, the influential factors under this study are "destination image, eWOM, attitude, subjective norm, and perceived behavioral control." Attitude, subjective norm and perceived behavioral control are the main constructs of the TPB model given by Ajzen [4].

The TPB is an extension of the previously defined concept of the theory of reasoned action (TRA) [6, 53]. In recent years, the TPB has become one of the most impactful and widely used models to study human behavior [157]. The TPB model is comprised of three primary components: "attitude, subjective norm, and perceived behavioral control" [4]. The TPB states that an individual's willingness to participate in a particular activity is determined by "attitude, subjective norm, and perceived behavioral control" [4]. Many studies have integrated the TPB model in the field of tourism and hospitality industry to predict travel intention [9, 14, 42, 69, 84, 91, 104, 108, 122, 135, 138, 139]. However, the phenomenon of predicting revisit intention post-COVID-19 is less studied. Hence, the present study bridges this gap by incorporating the TPB model along with additional constructs, viz. eWOM and destination image, and unwraps the role of eWOM in predicting revisit intention.

## Hypotheses development

### eWOM, attitude and revisit intention

Attitude (ATT) is one of the important components of the TPB model and is described as the positive or negative perception of certain behavior. Individuals with a more optimistic attitude are more likely to have a good behavioral intention and vice versa [155]. As a consequence, the theory of consumer decision-making emphasizes the importance of attitude [40], and it affects travelers' decision-making process favorably [73, 137]. Attitude is a key component that predicts, explains and influences travelers' behavioral intention [136] to engage in a certain behavior, such as travelling during or after a crisis or pandemic like the recent COVID-19 [105, 136]. Similarly, positive eWOM on social media influences behavioral intention. eWOM influences traveler’s decision-making process and motivates them for revisiting [87]. The findings of earlier studies show a positive association between eWOM, attitude and intention [17, 28, 61, 78, 79, 84, 134, 168]. Bilal et al. [29] found in their research that eWOM significantly and positively influences attitude. Yusuf et al. [170] also found a positive relationship between eWOM and attitude. Research by Avraham [19] shows that eWOM reduces tourists' risk perception in a crisis like situations like natural disasters, terror attacks and political disputes and enhances their intention to visit. Assaker and O'Connor [17] discovered that eWOM channels, including social media, travel review sites and online forums, were proven to be particularly helpful in reducing travelers' fears of political unrest and terrorist attacks when visiting a region. Hasan and Rahman [76] found a significant and positive association between attitude and green hotel revisit intention post-COVID-19. Sukaatmadja et al. [146], Hasan et al. [78], Liu et al. [102] and Suid et al. [143] also advocate a positive relationship between attitude and revisit intention. Since COVID-19 has significantly altered the psychology of tourists, it has become indispensable to re-examine the behavioral intention of tourists [172]. Developing positive attitude toward travelling post-COVID-19 is quite an intriguing task. The intention to travel will become stronger if a traveler has a positive attitude toward travelling post-COVID-19, and this growing intention leads to actual travelling. Hence, on the basis of evidence found in previous studies, the following hypotheses are postulated for the present study:

#### **H**_**1**_

eWOM positively influences attitude.

#### **H**_**2**_

Attitude positively influences revisit intention post-COVID-19.

### eWOM, subjective norm and revisit intention

Another important component of the TPB model is the subjective norm (SN) which is described as social influences that lead people to assume that a person (or group) approves and encourages specific behavior [68]. In terms of behavioral performance, SN may be described as "the perceived social pressure that is experienced" [156]. People who are important to a person if they suggest considering eWOM before finalizing a destination to visit in the future may be likely to direct their behavior toward performing a certain activity (in this case, toward considering eWOM about a destination). There has been a social pressure during the COVID-19 to follow the preventive measures, such as social isolation, regular hand washing and wearing a mask while travelling, to conform social accountability [20]. In that situation, if family or friends have a positive attitude toward travelling post-COVID-19, the traveler is more likely to travel to match their expectations, and vice versa. Jalilvand and Samiei [84] and Guoqing et al. [62] have found a positive relationship between eWOM and subjective norm. A significant association between eWOM and the subjective norm was also found by Lee et al. [96]. Bae and Chang [20] conducted a study to see the effect of COVID-19 risk perception on tourist behavioral intention and found a significant relationship between subjective norm and behavioral intention. Hasan and Rahman [76] conducted a study to understand the factors influencing green hotel revisit intentions after COVID-19 and found a significant positive association between subjective norm and revisit intention. In an another study conducted by Hasan [75], a significant association between subjective norm and behavioral intention was found. Also, previous studies show a positive association between subjective norm and revisit intention [1, 77, 123]. Hence, on the basis of evidence found in previous studies, the following hypotheses are postulated for the present study:

#### **H**_**3**_

eWOM positively influences subjective norm.

#### **H**_**4**_

Subjective norm positively influences revisit intention post-COVID-19.

### eWOM, perceived behavioral control and revisit intention

Perceived behavioral control (PBC) is regarded as an antecedent of intention and behavior [4]. According to Ajzen [4, 5], perceived behavioral control (PBC) refers to "the perceived ease or difficulty of performing the behavior," like a traveler considers how easy or difficult it is to travel during or after the COVID-19. When it comes to PBC, a person's belief control determines whether or not they have what they need to succeed [71, 157]. The PBC has an impact on consumer behavior and is somewhat within voluntary control [119]. However, studies on the socio-emotional nature generated out of technology-based communication [118, 154] demonstrated that given sufficient time, individuals might generate complete perceptions of others based only on the language of the written content of an e-message [31]. Suppose people have resources and the ability to reach an online WOM. In that case, they will likely get engaged in utilizing online content to satisfy the need for personal information requirements. In the context of COVID-19, PBC is the confidence a traveler has in his or her own ability to manage any situation that arises and any resources that are necessary for a safe and enjoyable trip. During the COVID-19, travel was restricted and people were unable to travel. Therefore, PBC is very important in the formation of travel-related behavioral intentions during or after COVID-19. Several studies indicate that self-confidence of an individual in their abilities to conduct a specific behavior is positively affected by their intention toward it [21, 38, 41]. Tourism destination choice is affected by visitors' perceptions of their ability to exert behavioral control over their travel options based on the content provided over social media, which is accessible to users [84]. Nguyen et al. [116] found a strong positive effect of PBC on travel intention post-COVID-19. Hasan [75] and Lee et al. [96] also found a significant association between PBC and behavioral intention. Some previous studies also found a positive association between PBC and revisit intention [1, 28, 77]. Hence, on the basis of evidence found in previous studies, the following hypotheses are postulated for the present study:

#### **H**_**5**_

eWOM positively influences perceived behavioral control.

#### **H**_**6**_

Perceived behavioral control positively influences revisit intention post-COVID-19.

### eWOM, destination image and revisit intention

Destination image is the mental depiction of an individual's knowledge (beliefs), emotions and general view of a certain destination [49]. According to Lee et al. [95], "destination image plays two important roles in behavior: to influence the destination choice decision-making process and to condition the after-decision-making behavior including participation (on-site experience), evaluation (satisfaction), and future behavioral intentions (intention to revisit)." The term "destination image" refers to a person's preconceived notions about a place based on their accumulation of knowledge about it gained from a variety of sources [57]. One of the very important and organic sources of information is word-of-mouth (WOM) which influences tourists' perception regarding a travel destination [85]. It is regarded as the most trustworthy and authentic source to get meaningful information [84]. The proliferation of the internet and information technology has made it possible for travelers to share their experiences of a destination online in the form of eWOM. eWOM is widely acknowledged as a significant information source that has a significant impact on travelers' decisions about their travel plans and destination preferences [84, 134, 168]. In the context of COVID-19, destination image plays a pivotal role in making travel-related decisions. The prevailing situation of COVID-19 at the particular destination strongly influences tourists’ decision to visit or not to visit that place. The positive the destination image the greater the intention to visit that place and vice versa. Previous studies found an association between eWOM and destination image [19, 48, 86, 130]. Ahmad et al. [3] conducted a study post-COVID-19 crisis recovery and found that destination image has a significant relationship with tourists' visit intention. Rasoolimanesh et al. [125] and Yang et al. [167] also found destination image as a strong determinant of revisit intention post-COVID-19 recovery phase. A positive association between destination image and revisit intention has also been identified in previous studies [1, 11, 93]. Hence, on the basis of evidence found in previous studies, the following hypotheses are postulated for the present study:

#### **H**_**7**_

eWOM positively influences destination image.

#### **H**_**8**_

Destination image positively influences revisit intention post-COVID-19.

### eWOM and revisit intention

The concept of behavioral intention is crucial to TPB because it reveals how an individual's intentions to perform or not to perform certain activity is reflected by their actions [4]. In addition, eWOM has been found to have an impact on people's purchasing decisions [84]. The term "eWOM" may be defined as "any positive or negative statement made by potential, actual, or former customers about a product which is made available to a multitude of people and institutes via the internet" [80]. Positive eWOM has been identified by developing a positive brand image and minimizing the sense of risk [87]. If a person comes across a positive eWOM, it is likely to increase visit intention. Intention to visit can be defined as the desire to visit a tourist destination by potential tourists [37]. eWOM communication has been captivating the attention of researchers in recent years [133], such as the impact on travel intention/behavior and destination selection [2, 84, 134]. Aktan et al. [7] conducted a study on global expats and found that expats' willingness to travel post-crisis was significantly influenced by eWOM. On the other hand, Zainuddin et al. [171] found that eWOM with other indicators positively impacts intention to visit natural places during COVID-19. A considerable number of tourism studies have indicated that electronic word-of-mouth (eWOM) may have an influence on travel intention [16, 50, 153]. Furthermore, it is undeniable that positive word-of-mouth (WOM) has a positive influence on travel intention [2]. During COVID-19, eWOM played a crucial role in the formation of travel-related decisions. People paid more attention to social media posts, online ratings and reviews, comments and shares over different online forums and communities, etc. Hence, on the basis of evidence found in previous studies, the following hypothesis is postulated for the present study:

#### **H**_**9**_

eWOM positively influences revisit intention post-COVID-19.

**Fig. 1:**
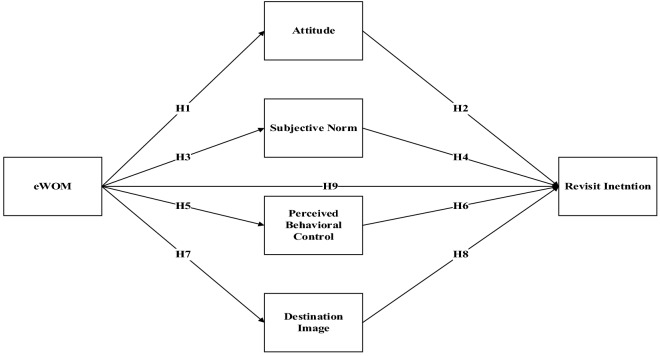
Theoretical framework. *Source*: The Authors

The hypotheses can be presented as shown in Fig. 1.

## Research methodology

### Research instrument

A well-structured online questionnaire was developed, followed by a comprehensive review of literature which was based on a 7-point Likert scale (where "1 = strongly disagree and 7 = strongly agree"). Seven-point Likert items have been shown to be more accurate, easier to use and a better reflection of a respondent's true evaluation [10, 52, 89]. Moreover, it has appeared that seven-point Likert scales are more appropriate for use with electronic dissemination [94]. Hence, a seven-point Likert scale was used in the present study. All the items used in this study were culled from highly referenced literature published in high-quality journals. The articulation and phrasing of the adapted items have been slightly changed to make them more relevant and suitable for the study. The whole questionnaire was divided into three parts. In the first part of the questionnaire, tourists were asked whether they had visited India in the past. If they had not visited India, their responses were rejected and vice versa. The second part comprised of questions related to the demographics of the respondents, i.e., gender, age, marital status, education, occupation and monthly income (INR). The third part of the questionnaire comprised questions measuring attitude, subjective norm, perceived behavioral control, revisit intention, electronic word of mouth and destination image. Sources of all the measurement items are given in Table 1.

**Table 1:** Items used in the questionnaire. *Source*: The Authors

Construct	No. of items	Source
ATT	4	Taylor and Todd [147]
SN	4	Ajzen [4], Venkatesh and Davis [151], Mathieson [109]
PBC	5	Taylor and Todd [147]
RI	4	Venkatesh et al. [152]
eWOM	4	Bambauer-Sachse and Mangold [22]
DI	3	Lee and Lockshin [97]

### Data collection

Data were collected via an online questionnaire on a convenience basis. This is a fast and simple sampling procedure [83, 144, 145]. Moreover, it is easy to contact a large number of audiences of similar interests using online surveys [72], which is otherwise tricky since finding and recognizing them is challenging [46]. A cross-sectional study approach was used in the present study. In this approach, data are collected from different individuals at one point in time [129]. Prior to distributing the questionnaire, it was decided to perform a pilot test with 50 answers to ensure that the questions and wordings were easy, clear and understandable to respondents. The pilot test rendered accurate and reliable results, and only after that the Google form link was deployed on social networking sites from February 1 to March 31, 2022. The reason behind choosing this period for data collection is that there was a significant drop both in the proportion of cases that were positive and the total number of active cases across the country [51, 149] and the country was moving to completely reopen all the activities [149]. The social media sites of travel agencies on which the questionnaire was circulated are among the popular travel agencies, namely Thomas Cook, Expedia, MakeMyTrip and Yatra.com [82]. The target population of the survey was tourists who follow the social media web pages of the travel agencies. There were 334 responses collected in total during the stipulated time period, but 33 responses were omitted due to missing values. Thus, for the final analysis, a total of 301 valid and useable responses were selected.

### Analysis

According to Anderson and Gerbing's [15] two-step procedure, CFA was first used to check the reliability and validity of the measurement model. One of the important reasons for using this procedure is that this CFA approach gives researchers comprehensive tools for evaluating and updating theoretical models [26, 33, 88]. After the measurement model was evaluated for adequacy, SEM was used to determine the model's suitability and to evaluate the hypotheses. This is due to the difficulty of directly examining many critical factors (latent variables) in social sciences, such as attitude, behavior, intention, motivation. [131]. Many indicators are used to measure these latent variables in SEM, which may contain measurement errors. As a result, SEM is the most critical instrument for assessing and analyzing the relationship between latent variables [35, 47] and is found suitable for the present study. The adequate sample size for using SEM should be 1:10 relative to the number of items in the questionnaire [65, 148, 163], and in the present study, there are 24 items in total. Therefore, the sample size of the present study meets this requirement and is adequate enough for using SEM. Data were analyzed using SPSS 20 and AMOS 22.0.

## Results

### Demographic profile of respondents

The demographic data depict that as much as 68.1% of the survey respondents are male, and 31.9% are female. The majority of the tourists (43.9%) come from the age group of 28–37. 53.5 percent of people are single, while 37.2 percent have acquired a graduate degree. The majority of the respondents are students (34.6%), and 27.9% are employed. As much as 31.9 percent of people's monthly income ranges between INR 15,001 and 30,000. At the time of the survey (on February 1, 2022), the exchange rate was 1 USD to INR = 74.763. This information was obtained from Exchange Rates UK. For detailed information, see Table 2.

**Table 2:** Respondent's profile (n = 301). *Source*: Primary Data

Demographic variable	Sub-variable	Frequency	Percent
Gender	Male	205	68.1
	Female	96	31.9
Age	Below 18	21	6.9
	18–27	85	28.2
	28–37	132	43.9
	38–47	39	13
	48–57	18	6
	Above 57	6	2
Marital status	Single	161	53.5
	Married	124	41.2
	Others	16	5.3
Education	Undergraduate	44	14.6
	Graduate	112	37.2
	Postgraduate	96	31.9
	Ph.D.	21	7
	Others	28	9.3
Occupation	Student	104	34.6
	Employed	84	27.9
	Retired	24	7.9
	Businessperson	58	19.3
	Others	31	10.3
Monthly Income (INR)	Upto 15,000	74	24.6
	15,001–30,000	96	31.9
	30,001–45,000	67	22.3
	45,001–60,000	35	11.6
	Above 60,000	29	9.6

### Descriptive statistics

The mean values of all the variables range from 4.3341 to 5.2479, and the standard deviations of all the variables fall between 1.08622 and 1.54373. Out of all the variables, revisit intention (RI) has the highest mean value (5.2479), and perceived behavioral control (PBC) has the lowest one (4.3341). PBC induces the highest standard deviation (1.54373), while attitude (ATT) shows the lowest one (1.08622). Detailed information is given in Table 3.

**Table 3:** Descriptive statistics. *Source*: Primary Data

Construct	Mean	SD
ATT	5.1988	1.08622
SN	4.5050	1.51551
PBC	4.3341	1.54373
RI	5.2479	1.53068
eWOM	5.0083	1.35536
DI	4.9240	1.47084

To explain the component structure and validate the scale, CFA was done using AMOS 22 [32]. Conceptually two types of validity, "convergent and discriminant," were tested using CFA. The convergent validity of a construct is defined as "the degree to which multiple different techniques of assessing a construct provide the same findings." Average Variance Extracted (AVE) was used to assess convergent validity, which is determined as the mean variance retrieved for the construct's loading. The value of AVE greater than 0.5 is acceptable. Composite reliability (CR) is another factor to consider, which is calculated using the square of each construct's total factor loading and total error variance terms [54, 64]. The CR has a threshold limit that is greater than 0.7 [43, 63, 106]. These limitations were reached in this study. Lastly, CR should be greater than AVE. As a result, all the conditions of convergent validity are satisfied. Detailed information is given in Table 4.

**Table 4:** Confirmatory factor analysis statistics. *Source*: Primary Data

Item	Factor loading	CR	AVE	Cronbach's α
*Attitude (ATT)*		0.931	0.771	0.921
ATT1: Making a destination choice on the basis of eWOM is a good idea	0.881			
ATT2: Making a destination choice on the basis of eWOM is a wise idea	0.850			
ATT3: I like the idea of eWOM while making a destination choice	0.836			
ATT4: Making a destination choice on the basis of eWOM would be a pleasant experience	0.850			
*Subjective norm (SN)*		0.973	0.902	0.973
SN1: People who influence my behavior would think that I should make a destination choice on the basis of eWOM	0.803			
SN2: People who are important to me think that I should make a destination choice on the basis of eWOM	0.802			
SN3: People whom I know would expect me to make a destination choice on the basis of eWOM	0.809			
SN4: People whose opinions I value prefer that I should make a destination choice on the basis of eWOM	0.751			
*Perceived behavioral control (PBC)*		0.971	0.870	0.971
PBC1: I would be able to make a destination choice on the basis of eWOM	0.838			
PBC2: Making a destination choice on the basis of eWOM is entirely within my control	0.869			
PBC3: I have the resources to make a destination choice on the basis of eWOM	0.891			
PBC4: I have the ability to make a destination choice on the basis of eWOM	0.884			
PBC5: I have the knowledge to make a destination choice on the basis of eWOM	0.869			
*Revisit intention (RI)*		0.951	0.828	0.950
RI1: I intend to make a destination choice on the basis of eWOM	0.757			
RI2: I predict I would make a destination choice on the basis of eWOM	0.829			
RI3: I plan to make a destination choice on the basis of eWOM	0.800			
RI4: I will strongly recommend others to make a destination choice on the basis of eWOM	0.747			
*Electronic word of mouth (eWOM)*		0.962	0.864	0.962
eWOM1: I often read other tourists' online travel reviews to know which destinations are safe to visit	0.842			
eWOM2: I frequently gather information from tourists' online travel reviews before I travel to a certain destination	0.861			
eWOM3: If I don't read tourists' online travel reviews when I travel to a destination, I worry about my decision	0.890			
eWOM4: When I travel to a destination, tourists' online travel reviews make me confident in travelling to the destination	0.882			
*Destination image (DI)*		0.955	0.876	0.954
DI1: India is safe and secure	0.764			
DI2: India offers exciting and interesting places to visit	0.781			
DI3: As a tourism destination, India offers good value for money	0.772			

The degree to which measures of distinct constructs are unique is referred to as discriminant validity [36]. Discriminant validity occurs "when the proportion of AVE in each dimension exceeds the square of the coefficient indicating its link to other dimensions" [54]. As per the results, the AVEs of all associated latent variables were greater than the square of the correlation between the constructs. As a result, the constructs in this study were found to have discriminant validity (Table 5).

**Table 5:** Discriminant validity. *Source*: Primary Data

	ATT	SN	PBC	RI	eWOM	DI
ATT	**0.878**					
SN	0.389	**0.950**				
PBC	0.330	0.667	**0.933**			
RI	0.369	0.670	0.493	**0.910**		
eWOM	0.357	0.466	0.509	0.658	**0.930**	
DI	0.561	0.710	0.483	0.423	0.416	**0.936**

*p* < 0.001; square root of AVE diagonally highlighted in bold

In accordance with the first CFA results, the following indices showed that the model fit was adequate: (χ^2^/*df* = 3.57, RFI = 0.901, NFI = 0.916, CFI = 0.938, GFI = 0.812, TLI = 0.927, IFI = 0.938, RMSEA = 0.073). The value of RMSEA shows a mediocre fit but is acceptable, as per Browne and Cudeck [34]. The suggested value for such indices should be more than 0.90; however, some values greater than 0.80 are also acceptable [25, 45, 114]. See Table 6 for additional information.

**Table 6:** SEM fit indices. *Source*: Primary data

Fit indices	Cut off values	Model study	References
Absolute fit measure			Marsh and Hocevar [107], Hair et al. [65], Raykov and Marcoulides [126], Harrington [74], Schumacker and Lomax [127]
CMIN/DF	≥ 5	3.57	
RMSEA	< 0.05, < 0.08	0.073	
Incremental fit measure			
CFI	> 0.90	0.938	
TLI	> 0.90	0.927	
GFI	> 0.80	0.812	
NFI	> 0.90	0.916	
IFI	> 0.90	0.938	
RFI	> 0.90	0.901	

### Hypotheses testing

**Fig. 2:**
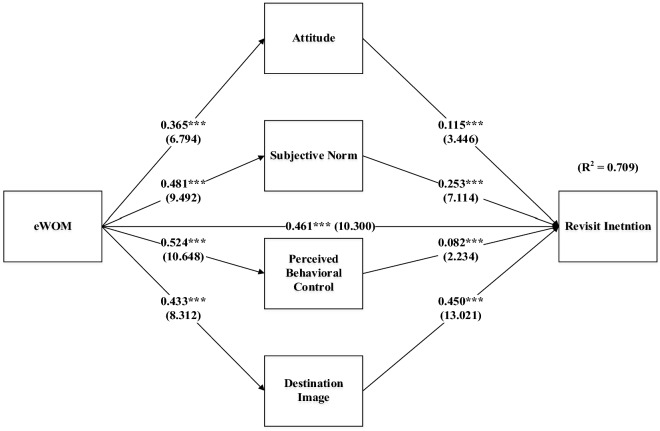
Structural model and hypotheses. *Source*: AMOS Output

CFA was performed by the researchers prior to testing the hypotheses that were suggested for the study in order to verify the validity and reliability of the instrument. Figure 2 depicts the path coefficient, which shows how one hypothesis is connected to the other. It is noticed that all nine hypotheses support the results and are thus accepted. eWOM significantly and positively influences ATT (*β* = 0.365, *t* value = 6.794, *p* < 0.001), SN (*β* = 0.481, *t* value = 9.492, *p* < 0.001), PBC (*β* = 0.524, *t* value = 10.648, *p* < 0.001) and DI (*β* = 0.433, *t* value = 8.312, *p* < 0.001). It is also evident from the findings that eWOM has a positive and significant influence on revisit intention post-COVID-19 (*β* = 0.461, *t* value = 10.300, *p* < 0.001). The core constructs of TPB that are ATT (*β* = 0.115, *t* value = 3.466, *p* < 0.001), SN (*β* = 0.253, *t* value = 7.114, *p* < 0.001), PBC (*β* = 0.082, *t* value = 2.234, *p* < 0.001) also significantly and positively influence revisit intention post-COVID-19. In addition, destination image also has a positive and significant influence on revisit intention post-COVID-19 (*β* = 0.450, *t* value = 13.012, *p* < 0.001). Hence, all nine hypotheses, H_1_ to H_9,_ are supported by the evidence.

The results reveal that all the constructs under study, viz. "electronic word of mouth (eWOM), destination image (DI), attitude (ATT), subjective norm (SN) and perceived behavioral control (PBC)" are significant in predicting "revisit intention" post-COVID-19. These constructs explained approx. 71% (*R*^2^ = 0.709) of the variance in the revisit intention post-COVID-19 (Table 7).

**Table 7:** Summarized hypotheses. *Source*: AMOS Output

Relationship	Std. *β*	*t* value	Results
ATT ← eWOM	0.365	6.794	Supported
SN ← eWOM	0.481	9.494	Supported
PBC ← eWOM	0.524	10.648	Supported
DI ← eWOM	0.433	8.312	Supported
RI ← ATT	0.115	3.446	Supported
RI ← SN	0.253	7.114	Supported
RI ← PBC	0.082	2.234	Supported
RI ← DI	0.450	13.021	Supported
RI ← eWOM	0.461	10.300	Supported

## Discussion and conclusion

The aim of the present study was to re-examine the impact of social media eWOM on revisit intention post-COVID-19 incorporating destination image with the TPB model. In order to evaluate the effectiveness of the measuring model for reflective constructs, the present research investigated the criteria of internal reliability, convergent validity and discriminant validity [67]. After assessing the validity of the measurement model, the current study employed the structural model to evaluate the hypotheses. The proposed model explained 71% (*R*^2^ = 0.709) of the variance in the revisit intention post-COVID-19, which was higher than the minimum threshold value of *R*^2^ = 25% [66]. Thus, the predictive relevance of the model is very high. Additionally, nonparametric bootstrapping with 5,000 replicates was employed to test the proposed hypotheses [66]. Consequently, all nine hypotheses, H_1_ to H_9_, were supported in terms of the direct effect.

Since the "user-generated content" (UGC), travelers now have the ability to obtain and disseminate travel and tourism-related information. Peer-generated information related to tourist destinations over social media channels is now readily available to travelers throughout the world. Keeping in view the growing significance of social media, the prime objective of the present study was to reinvestigate the influence of eWOM on tourists' revisit intention post-COVID-19. The relevance of incorporating destination image and eWOM with the TPB model lies in the fact that positive eWOM over social media lessens the stress of travelers in choosing a tourism destination post-COVID-19. In the wake of COVID-19, travelers are cautious about their safety and security [144, 145]. They are more concerned about becoming contaminated. Travel is restricted, and passengers perceive a higher risk of disease transmission while traveling [69]. In this mayhem, a positive eWOM about a tourism destination may help them in choosing a safe and secure destination. Hence, the present study highlighted the significance of social media eWOM and destination image in analyzing the tourists' revisit intention post-COVID-19.

The TPB served as the foundation for this study, which examined tourists' revisit intention by extending the TPB original model with the inclusion of eWOM and destination image. The results of the study significantly support the TPB model in revisiting a tourism destination post-COVID-19. As a result, theoretically and empirically, authors have effectively extended the original TPB in accordance with Ajzen's [4] criteria for enhancing the theory. To the best of researchers' knowledge, no empirical work has been done taking the studied variables to re-examine the effect of social media eWOM on revisit intention post-COVID-19 in the Indian context.

The findings of the study revealed that the effect of eWOM on revisit intention was the strongest among all the constructs, followed by destination image. This finding is in line with previous studies [18, 50, 84, 121, 153]. The main components of the TPB model: "attitude, subjective norm and perceived behavioral control," are found to have a significant and positive influence on tourists' revisit intention. ATT (*β* = 0.115, *t* value = 3.466, *p* < 0.001) significantly and positively influences revisit intention. This finding is in line with previous studies [79, 110, 123, 136]. Consequently, it is implied that tourists have a rosy outlook on revisiting India post-COVID-19. In addition, Sparks [138] defined it a pull-related belief that helps one to maintain an optimistic attitude toward revisit intention. Hence, it is implied that tourists have an optimistic attitude for India and they intend to revisit India post-COVID-19. PBC (*β* = 0.082, *t* value = 2.234, *p* < 0.001) also has a significant and positive effect on revisit intention. This finding is in line with previous studies [44, 98]. In this study 68.1% of the respondents are male, 43.9% come from the age group of 28–37 and 53.5% are single. They have proper education and income to make their travel decisions. Hence, it is implied that tourists do not perceive any difficulty, viz. financial or any other, to revisit India. Among all the three core constructs of the TPB, subjective norm came out as the most influential and the strongest predictor of revisit intention (*β* = 0.253). These findings are in line with the previous studies [58, 70, 111, 120, 123]. The reason for this could be that post-COVID-19, people are more inclined toward the actions and words of others because of the agility of COVID-19-related information. Another reason for this could be that people are more tend to referent others while making decisions. The collectivist cultural approach dominates the decision-making process. In this study, a significant chunk of the respondents are students. Students get easily influenced by others. So this could be another possible reason of subjective norm as a significant predictor of revisit intention in the present study.

Besides the core constructs of the TPB, social media eWOM came out as the most influential and strongest predictor of revisit intention (*β* = 0.461). This is in continuation with the findings of Jalilvand and Samiei [84]. The reason for this could be that to make the revisit decisions post-COVID-19, tourists are more inclined to social media eWOM for choosing a safe and secure destination. Online ratings and reviews, referrals and recommendations, online forums and communities play a significant role in formulating behavioral intention. Tourists pay more attention to social media eWOM when it comes to choose a safe and secure destination. Therefore, eWOM came out the strongest predictor of revisit intention post-COVID-19. Destination image is the second most influential and strongest predictor of revisit intention (*β* = 0.450) after the eWOM. These findings are in line with the previous studies [18, 121]. Tourists are more cautious in choosing a destination post-COVID-19 due to the ongoing COVID-19 situation at the chosen destination, the travel-related relaxations, government visa policies, etc. This could be the possible reason of destination image came out to be the second strongest predictor of revisit intention. The findings of the present study are different from previous studies in the sense that among all the constructs, eWOM came out the strongest and most influential predictor of revisit intention followed by destination image. The positive the eWOM the stronger will be the image of a destination and the higher will be the revisit intention.

## Implications

### Theoretical implications

The present study extends a profound theoretical backing and contributes significantly to the existing stock of literature on the TPB, social media eWOM and tourists' revisit intention post-COVID-19. The theoretical framework proposed in the present study gives meaningful insights to academicians and researchers to understand the role of eWOM in revisiting tourism destinations in the post-COVID-19 period. This study empirically tests and validates the TPB model in predicting tourists' revisit intention post-COVID-19 by incorporating destination image and eWOM. The inclusion of these additional constructs improves the robustness and predictive ability of the proposed theoretical framework while assessing tourists' revisit intention post-COVID-19. This study adds value to the existing literature in many ways. First and foremost, it contributes to the literature on social media eWOM and its role in travel and tourism post-COVID-19. As far as authors are concerned, no study has been carried out that has measured the effect of eWOM and destination image on revisit intention incorporating TPB in the post-COVID-19 period and that too in the Indian context. Thus, the present study is very crucial as it bridges this gap by providing a strong theoretical foundation for future researchers and academicians. Second, the findings confirm the relevance of destination image and eWOM in determining the tourists' revisit intention post-COVID-19 and improve the understanding of the subject matter. In addition, the extended model gives an evocative explanation of the main drivers of behavioral intention that will aid future researchers and academicians in better understanding the various components affecting it.

### Practical implications

This research also has some practical implications for travel and tourism companies, tourism destination marketers, industry practitioners and social media web developers. It is evident from the findings of the study that social media has now become an indispensable part of exchanging travel-related information and experience. Therefore, travel and tourism companies and service providers must register their strong presence on social media to better understand their customer base, cater for their emerging needs and make close relations with potential customers. By doing so, they would have positive word of mouth and could gain a competitive advantage over their counterparts. The findings revealed that eWOM significantly and positively influences the revisit intention of tourists. Therefore, tourists should be encouraged to share positive eWOM relating to the business or service provided to them. Hence, online marketing managers should pay close attention to this area of focus. In this regard, travel and tourism-related service-providing companies must focus on getting quick feedback about their offerings to improve the quality of their services. Social media web developers can also be benefited from the findings of this study. They must design the web pages in such a way that quick and easy access to required information would be feasible. Sharing, gathering and disseminating travel-related information and search could take place in a matter of seconds. The process of feedback must be the quickest and easiest so that travel and tourism companies can come to know about the demands of their potential customers and can provide services accordingly.

This study has implications for administrators of tourist attractions all around the world who want to influence tourists' revisit intention. The competitive advantage of tourism destinations may be improved by developing methods to build positive eWOM, optimistic attitude and positive destination image experienced by visitors by increasing the value of their experience and the standard of the service they get, with the goal of increased revisit intention. In the light of the findings of the present study, which indicate that attitude, subjective norm and perceived behavioral control have a significant positive effect on revisit intention, travel and tourism companies, tourism destination marketers and industry practitioners must understand the strong influence of these determinants and try to take advantage out of them in forming tourists' revisit intention.

In addition, the favorable influence of destination image on revisit intention underscores the significance of tourism destination spots. The whole travel experience of tourists may be negatively impacted by a single occurrence, which may result in increased levels of discontent and a decreased desire to return to the destination. It is the responsibility of the managers of tourist attractions as well as the service providers to ensure that frontline workers maintain excellent levels of service to arriving visitors. As a consequence of this, service providers should place a greater emphasis on the provision of training that motivates staff to give exceptional service to clients and to create a favorable image of their location. In addition, the image of a location depends on several practical criteria, such as the availability of tourist amenities, safety, security and cleanliness. Therefore, a positive image encourages tourists to make repeat visits, whereas dissatisfied and unhappy tourists may not return to a destination, despite the fact that they have a positive impression of the destination's image, mainly when the destination's practical aspects are subpar in comparison with those of other locations. As a result, in order to improve the image of the destination, both local companies and the government may contribute to the development of a favorable image of the destination from the perspective of visitors.

## Limitations and future research directions

No study is complete without limitations and the present one is no exception. Although meeting its objective and rendering several theoretical and practical implications to academicians and industry practitioners, the study still has certain limitations that will pave the ground for future research. First, this study was executed in India. Being a developing country, it has a huge impact on referent others on behavioral intention. As a result, future research could be conducted in other developed countries for more robust results and generalization. Second, the TPB model was tested and validated in this study using eWOM and destination image in the context of COVID-19. Future studies could incorporate more determinants to measure behavioral intention, and their compound effect could be assessed using other behavioral theories. Third, data were obtained from 301 respondents due to time constraints. Future studies could be carried out using a large sample size. Fourth, the conclusions of this study were based on a survey conducted by the researchers. A qualitative technique might be used to acquire a more in-depth knowledge of the aspects examined in this study, which would be beneficial to future researchers. Fifth, the nature of the current research is cross-sectional in design. Researchers in the future might gather data on a longitudinal basis in order to get more enhanced and accurate results. Sixth, the convenience sampling method was used to gather the data in this study; however, researchers in the future may attempt to use a sampling method that is different from the one used here. Finally, the proposed framework suggested in this research could be evaluated in a variety of contexts other than tourism. Hence, the results could be varied.

## Data Availability

The authors declare that all types of data used in this study are available for any clarification. The authors of this manuscript are ready for any justification regarding the dataset. To make available of the dataset used in this study, the seeker must mail to the mentioned email address. The profile of the respondents is completely confidential.
